# Molecular floating-gate single-electron transistor

**DOI:** 10.1038/s41598-017-01578-7

**Published:** 2017-05-08

**Authors:** Makoto Yamamoto, Yasuo Azuma, Masanori Sakamoto, Toshiharu Teranishi, Hisao Ishii, Yutaka Majima, Yutaka Noguchi

**Affiliations:** 1 School of Science and Technology, Meiji University, Kawasaki, 214-8571 Japan; 2 Laboratory for Materials and Structures, Tokyo Institute of Technology, Yokohama, 226-8503 Japan; 3 Institute for Chemical Research, Kyoto University, Kyoto, 611-0011 Japan; 4 Center for Frontier Science, Chiba University, Chiba, 263-8522 Japan

## Abstract

We investigated reversible switching behaviors of a molecular floating-gate single-electron transistor (MFG-SET). The device consists of a gold nanoparticle-based SET and a few tetra-tert-butyl copper phthalocyanine (ttbCuPc) molecules; each nanoparticle (NP) functions as a Coulomb island. The ttbCuPc molecules function as photoreactive floating gates, which reversibly change the potential of the Coulomb island depending on the charge states induced in the ttbCuPc molecules by light irradiation or by externally applied voltages. We found that single-electron charging of ttbCuPc leads to a potential shift in the Coulomb island by more than half of its charging energy. The first induced device state was sufficiently stable; the retention time was more than a few hours without application of an external voltage. Moreover, the device exhibited an additional state when irradiated with 700 nm light, corresponding to doubly charged ttbCuPc. The life time of this additional state was several seconds, which is much shorter than that of the first induced state. These results clearly demonstrate an alternative method utilizing the unique functionality of the single molecule in nanoelectronics devices, and the potential application of MFG-SETs for investigating molecular charging phenomena.

## Introduction

Organic molecules are ideal candidates for bottom-up electronics components because of their atomically controlled structures and functionalities^1–4^. The charge, spin, and thermal transport characteristics of individual single molecules in a molecular junction (metal–molecule–metal) have been investigated via scanning probe microscopes^5–9^, mechanically controllable break junctions^10, 11^, and nanogap electrodes^12, 13^. Although these previous studies revealed the transport mechanisms of single molecules in molecular junctions, the fabrication of single-molecular devices is still challenging. One of the main difficulties in this field is the formation of molecular junctions in solid-state device structures. Moreover, the charge states of single molecules have not been sufficiently studied for charge transport through single molecules, even though understanding them would provide significant benefits for single-molecular devices^14, 15^.

Chemically synthesized metal or semiconductor NPs have also attracted much attention because of their potential applications in bottom-up electronics^16–18^. AuNPs are ideal materials for the fabrication of Coulomb islands owing to their uniform density of states, and large charging energy. AuNP-based SETs show clear Coulomb diamonds, which can be well-characterized by the orthodox theory^19–25^. Moreover, flexible logic operations were demonstrated in chemically assembled double-gate AuNP-SETs^26^.

Recently, we proposed a new device structure, *i*.*e*., molecular floating-gate SETs (MFG-SETs) in which individual molecules function as floating gates^27–30^. Organic molecules are smaller than typical NPs; thus, several functional molecules can be attached on an NP, which functions as a Coulomb island. Since SETs are extremely sensitive to the charge distribution adjacent to the Coulomb island^21, 31–33^, changes in the charge state of the organic molecules can affect the SET characteristics. Thus, we propose that the molecular floating gate allows for advanced functions of SETs without the need for multiple gate electrodes. Moreover, various single molecular functionalities can be utilized in solid-state device structures without forming typical molecular junctions.

In previous studies, we reported photoinduced conductance switching behaviors in copper phthalocyanine (CuPc)-doped metal NP SETs. The photoresponse of these devices originated in the photoinduced charging nature of CuPc, which is enhanced over the absorption range of CuPc. The charge state of CuPc changes the potential of the Coulomb island, which results in the gate offset of the SET; *i*.*e*., CuPc functions as a floating gate in the device. Photoinduced molecular charging involves three processes^27–29^; (i) exciton formation in the molecule by light absorption, (ii) exciton dissociation and charge transfer to the outside of the molecule (*e*.*g*., Coulomb island, source, drain, gate electrodes), (iii) stabilization of the charged molecule. The stability of the charge state depends on the local electrostatic environment^34^. Interestingly, when a silver nanoparticle (AgNP) was used as a Coulomb island, the polarity of the potential shift depended on the wavelength of the incident light, *i*.*e*., the gate offset shift was negative for irradiation with 500-nm light and positive with 600-nm light. The negative potential shift suggests that there was electron transfer from the AgNPs to the doped molecule–possibly via the localized surface plasmon resonance of AgNPs^30^.

In our previous works, we revealed the photoinduced switching behaviors in the nanoparticle-based SETs doped with CuPc molecules. However, the structure of those devices was not well defined, *i*.*e*., the stability diagram exhibited nonuniform Coulomb diamonds, implying the charge transport through several nanoparticles. This situation made the quantitative analyses of the molecular floating effect difficult, since we could not determine the numbers of nanoparticles, the charging energy of each nanoparticles, the nanoparticles coupled with molecules and so on.

For further understanding the molecular floating gate effect, a single-dot SET doped with isolated molecules were required. In this study, we employed an electroless gold plating method for the nanogap formation instead of the conventional electromigration method to improve the device yield. We successfully obtained a well-defined single-dot SET, which is satisfactory to the quantitative analysis of the molecular-floating gate effect. We found the potential shift in the Coulomb island originating from single-electron charging of tetra-tert-butyl CuPc (ttbCuPc) was approximately 50 meV, which was larger than half the charging energy of the Coulomb island (73 meV). The device state induced by singly charged ttbCuPc was sufficiently stable for more than a few hours without application of an external voltage. Moreover, the device exhibited an additional state with a short life time of several seconds under 700-nm light irradiation. We assigned this additional state to doubly charged ttbCuPc.

## Results and Discussion

**Figure 1:**
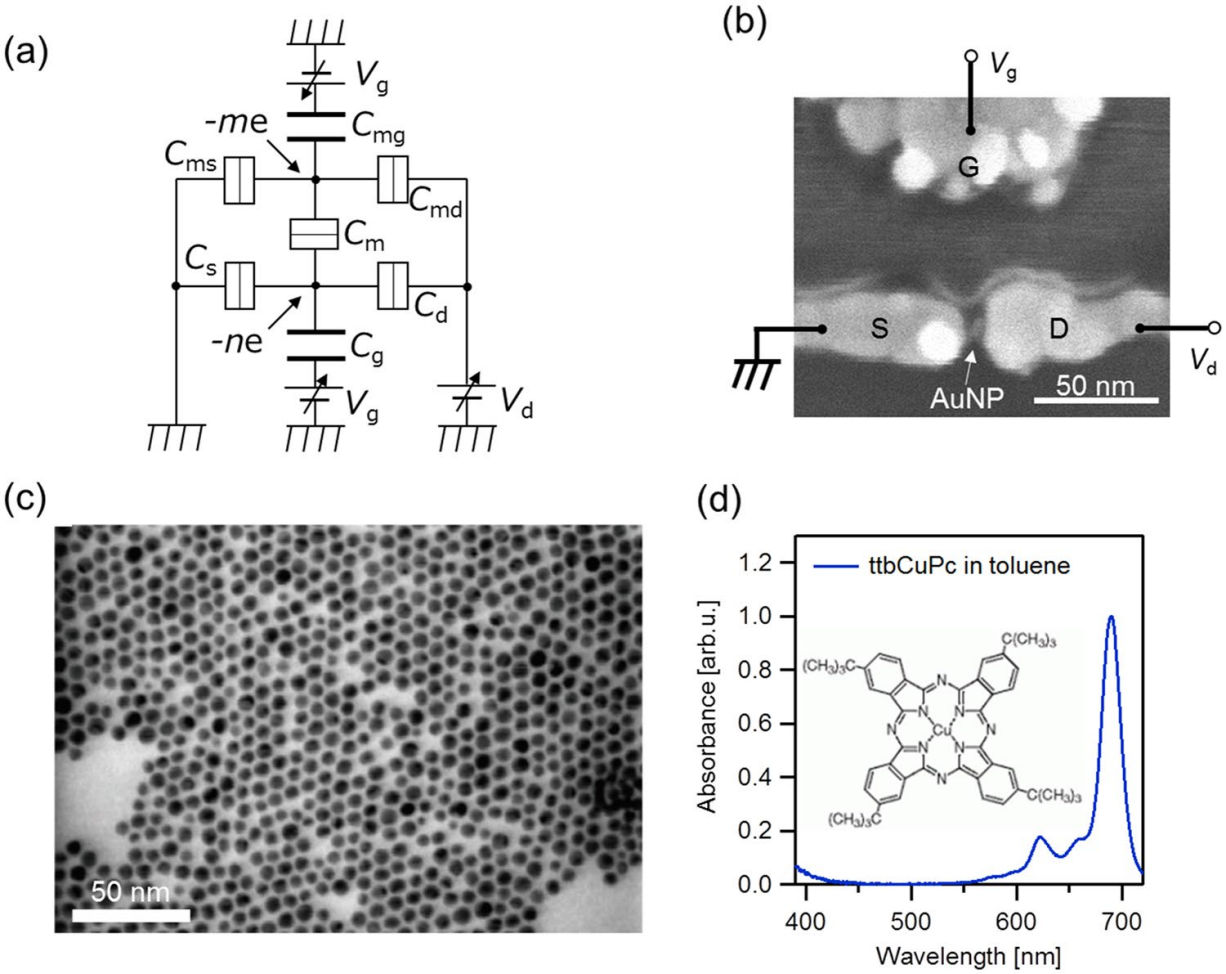
(**a**) Equivalent circuit of MFG-SET. (**b**) SEM image of MFG-SET. The bright spot between the source (S) and drain (D) electrodes is AuNP, which functions as a Coulomb island. (**c**) TEM image of AuNPs used in this study. (**d**) Absorption spectrum of ttbCuPc molecule in a toluene solution. The inset figure shows the molecular structure.

Using an equivalent circuit model (Fig. 1(a)), we study the fundamental properties of MFG-SETs. Our device consists of an SET and a molecular floating gate. The capacitance of each junction is defined as shown in Fig. 1(a). The numbers of electrons on AuNP and MFG are *n* and *m*, respectively. Tunneling junctions are formed among the Coulomb island, MFG, and source/drain electrodes.

The potentials of the Coulomb island (*V*_NP_) and MFG (*V*_mol_) at certain drain and gate voltages (*V*_d_ and *V*_g_, respectively) are given by

1 $${V}_{{\rm{N}}{\rm{P}}}=\frac{1}{{C}_{{\rm{m}}}}\frac{\chi }{1-\chi \eta }[({C}_{{\rm{d}}}+\eta {C}_{{\rm{m}}{\rm{d}}}){V}_{{\rm{d}}}+({C}_{{\rm{g}}}+\eta {C}_{{\rm{m}}g}){V}_{{\rm{g}}}-(n+\eta m)e],$$

2 $${V}_{{\rm{m}}{\rm{o}}{\rm{l}}}=\frac{1}{{C}_{{\rm{m}}}}\frac{\eta }{1-\chi \eta }[({C}_{{\rm{m}}{\rm{d}}}+\chi {C}_{{\rm{d}}}){V}_{{\rm{d}}}+({C}_{{\rm{m}}{\rm{g}}}+\chi {C}_{{\rm{g}}}){V}_{{\rm{g}}}-(m+\chi n)e].$$

Here, *η* and *χ* indicate the degree of electrostatic interaction between AuNP and MFG, *i*.*e*.,

3 $$\chi =\frac{{C}_{{\rm{m}}}}{{C}_{{\rm{\Sigma }}}+{C}_{{\rm{m}}}},$$

4 $$\eta =\frac{{C}_{{\rm{m}}}}{{C}_{{\rm{m}}{\rm{\Sigma }}}+{C}_{{\rm{m}}}},$$

where ${C}_{{\rm{\Sigma }}}={C}_{{\rm{s}}}+{C}_{{\rm{d}}}+{C}_{{\rm{g}}}$ and ${C}_{{\rm{m}}{\rm{\Sigma }}}={C}_{{\rm{ms}}}+{C}_{{\rm{md}}}+{C}_{{\rm{mg}}}$. The charging energy of a AuNP (*E*_c_) can be obtained according to

5 $$\begin{array}{ccc}{E}_{{\rm{c}}} & = & e[{V}_{{\rm{N}}{\rm{P}}}(n+1)-{V}_{{\rm{N}}{\rm{P}}}(n)]\\  & = & \frac{1}{{C}_{{\rm{m}}}}\frac{\chi {e}^{2}}{1-\chi \eta }.\end{array}$$

Similarly, the charging energy of MFG (*E*_mc_) can be described by

6 $$\begin{array}{rcl}{E}_{{\rm{mc}}} & = & e[{V}_{{\rm{mol}}}(m+1)-{V}_{{\rm{mol}}}(m)]\\  & = & \frac{1}{{C}_{{\rm{m}}}}\frac{\eta {e}^{2}}{1-\chi \eta }\mathrm{.}\end{array}$$

Thus, we obtain

7 $$\frac{{E}_{{\rm{mc}}}}{{E}_{{\rm{c}}}}=\frac{\eta }{\chi }\mathrm{.}$$

Generally, the capacitance of a single molecule is much smaller than that of a AuNP; therefore, we can assume that *η* ≫ *χ* (*E*_mc_ ≫ *E*_c_,) and that the main current path from the source to the drain electrode runs through the AuNP.

*V*_NP_ depends on the number of electrons on MFG. When MFG is charged by one excess electron, the potential shift in the Coulomb island (Δ*E*_mfg_) is

8 $$\begin{array}{ccc}{\rm{\Delta }}{E}_{{\rm{m}}{\rm{f}}{\rm{g}}} & = & e[{V}_{{\rm{N}}{\rm{P}}}(m+1)-{V}_{{\rm{N}}{\rm{P}}}(m)]\\  & = & -\frac{1}{{C}_{{\rm{m}}}}\frac{\chi \eta {e}^{2}}{1-\chi \eta }.\end{array}$$

At *V*_d_ = 0, the condition for adding one electron from the source or drain electrode to the Coulomb island is *eV*_NP_ ≥ *E*_c_/2; therefore,

9 $${V}_{{\rm{g}}}\ge \frac{\mathrm{(1/2}+n+\eta m)e}{{C}_{{\rm{g}}}+\eta {C}_{{\rm{mg}}}}$$

must be satisfied. The period of Coulomb oscillation along the gate voltage axis is

10 $${\rm{\Delta }}{V}_{{\rm{g}}}=\frac{e}{{C}_{{\rm{g}}}+\eta {C}_{{\rm{mg}}}}\mathrm{.}$$

The gate voltage offset due to one electron charging of MFG (Δ*V*_mfg_) is similarly obtained according to

11 $${\rm{\Delta }}{V}_{{\rm{m}}{\rm{f}}{\rm{g}}}=\frac{\eta e}{{C}_{{\rm{g}}}+\eta {C}_{{\rm{m}}{\rm{g}}}}.$$

Therefore, *η* can be experimentally determined according to

12 $$\begin{array}{ccc}\eta  & = & \frac{{\rm{\Delta }}{V}_{{\rm{m}}{\rm{f}}{\rm{g}}}}{{\rm{\Delta }}{V}_{{\rm{g}}}}\\  & = & -\frac{{\rm{\Delta }}{E}_{{\rm{m}}{\rm{f}}{\rm{g}}}}{{E}_{{\rm{c}}}}.\end{array}$$

Here, *η* corresponds to the efficiency of the floating-gate effect, which is dependent on the potential shift in the Coulomb island induced by a single additional electron on MFG. At maximum efficiency (*η* = 1), Δ*E*_mfg_ equals *E*_c_. Conversely, there is no potential shift in the Coulomb island for *η* = 0.

Our MFG-SETs consist of a nanogap electrode with side gate electrodes, a AuNP, and ttbCuPc molecules. Figure 1(b) shows a scanning electron microscope image of the measured MFG-SET.

AuNPs with a core diameter of 6.2 ± 0.8 nm, covered with decanethiols, are used as Coulomb islands. Figure 1(c) shows a transmission electron microscopy (TEM) image of one of the AuNPs used in this study. Here, ttbCuPc molecules are used for the molecular floating gate^30^. The absorption spectrum of ttbCuPc in a toluene solution is shown in Fig. 1(d). There are about 7 ttbCuPc molecules per one AuNP (see Fabrication process); however, as the bulky tertiary butyl groups prevent molecular aggregation, we expect the isolated CuPc core to function as a floating gate^35^.

**Figure 2:**
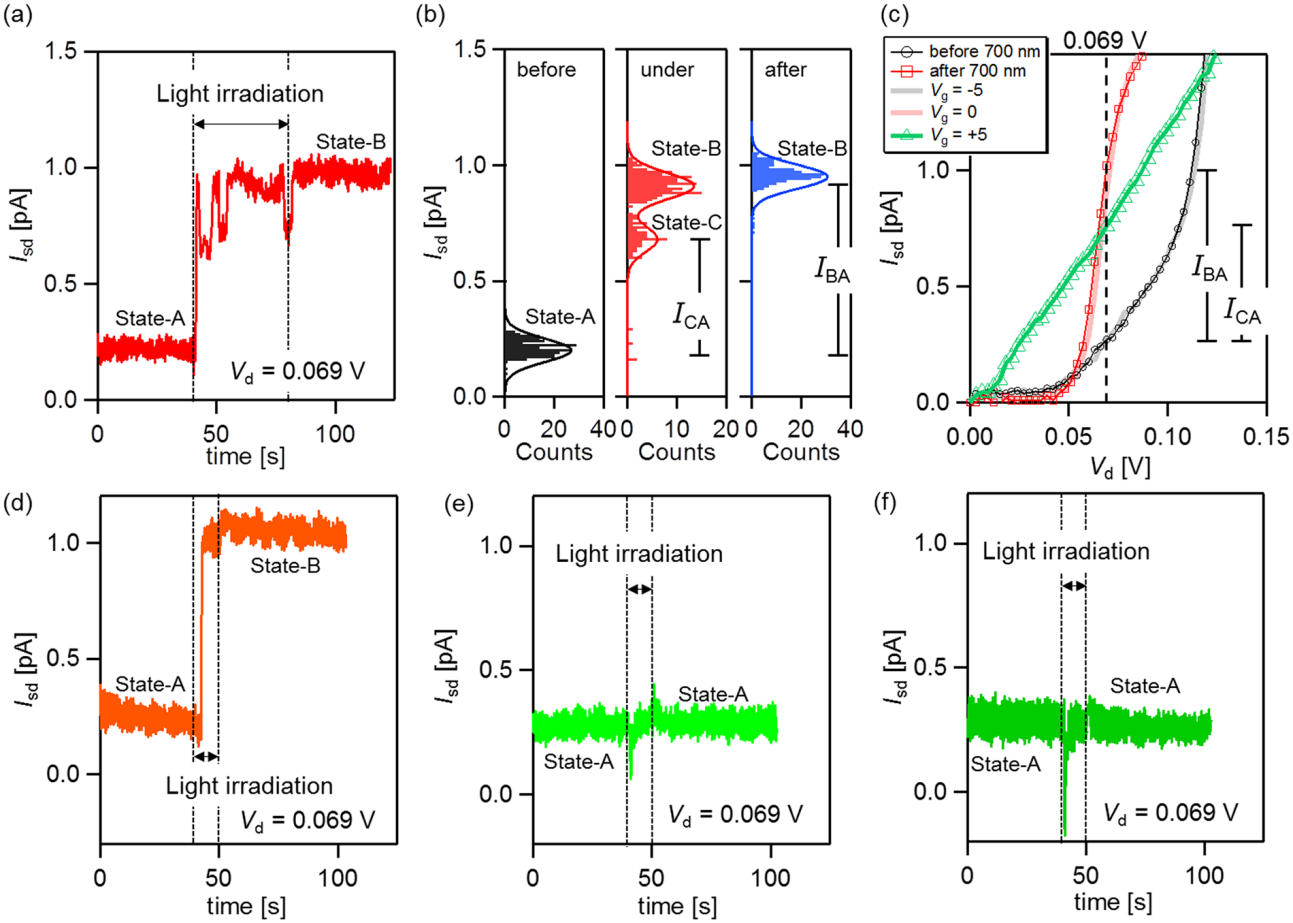
*I*_sd_ − *t* characteristics before, under, and after light irradiation (*V*_d_ = 0.069 V) at (**a**) 700 nm, 280 *μ*W/mm^2^; (**d**) 600 nm, 52 *μ*W/mm^2^; (**e**) 520 nm, 128 *μ*W/mm^2^; and (**f**) 500 nm, 79 *μ*W/mm^2^. (**b**) Histograms of the current before, under, and after 700-nm light irradiation. Each fitting curve comprises Gaussian functions. (**c**) *I*_sd_ − *V*_d_ characteristics before and after irradiation with 700-nm light and *V*_g_ = −5, 0, 5 V.

Figure 2(a,d–f) shows the current–time (*I*_sd_ − *t*) curves before, under, and after light irradiation with a constant drain voltage (*V*_d_ = 0.069 V).

As shown in Fig. 2(a), the current intensity is stable around 0.20 pA prior to irradiation with 700-nm light. When the device is illuminated, the current intensity increases to a higher level (around 0.91 pA), which remains even after the light irradiation ceases. Note that the change persists until high gate voltage (*V*_g_ > 8.8 V) is applied. In addition, the current intensity sometimes decreases to ~0.69 pA when irradiated at 700 nm. Figure 2(b) represents the histograms of the current before, under, and after 700-nm light irradiation, and each fitting curve comprises Gaussian functions. A single peak appears in the histogram before and after light irradiation at 0.20 pA and 0.95 pA, and we refer to these device states as state A and state B, respectively. Further, the histogram splits into two peaks under the irradiation, with peak current intensities of 0.91 pA and 0.68 pA. The first of these current peaks (0.91 pA) is almost the same as that after light irradiation has ceased (0.95 pA). The second peak originates from the current drops observed in Fig. 2(a), indicating that the device is in another state (state C). State C is relatively unstable and only appears under light irradiation with 700-nm light. The averaged lifetime of state C is several seconds.

Figure 2(c) shows *I*_sd_ − *V*_d_ characteristics before and after 700 nm light irradiation. A zero current region caused by a Coulomb blockade is observed in each *I*_sd_ − *V*_d_ curve. The black curve in Fig. 2(c) shows the *I*_sd_ − *V*_d_ characteristics before light irradiation (state A); this curve agrees well with the *I*_sd_ − *V*_d_ curve at *V*_g_ = −5 V measured after light irradiation has ceased (state B). Similarly, the red curve shows the *I*_sd_ − *V*_d_ characteristics after light irradiation has ceased at *V*_g_ = 0 V (state B). Thus, light irradiation induces a negative gate offset (Δ*V*_mfg_ = −5 V), indicating that MFG is positively charged (Equation (11)). Moreover, the current intensity of state C agrees well with that of state B at *V*_g_ = +5 V (green curve in Fig. 2(c)), indicating that MFG is further charged positively. Here, the first induced device state (state B) is sufficiently stable; the retention time is more than a few hours without the application an external voltage while that of state C is several second.

The photoinduced transition from state A to state B can also be induced with 600-nm light, while it can not be induced at wavelength of 500 and 520 nm. These results are consistent with those of a previous study^29^, and are in agreement with predictions based on the absorption spectrum of ttbCuPc, which has a finite absorbance at 600 and 700 nm, while displaying very little absorbance at 500 and 520 nm (Fig. 1(d)). In addition, the photoinduced transition does not depend on the intensity of the incident light; the intensity of the 600-nm light is weaker than that of the 500- and 520-nm light. Thus, we conclude that the photoinduced change is initiated by the light absorption of the ttbCuPc molecule.

Additionally, transient current is initially observed with 500- and 520-nm light irradiation (Fig. 2(e),(f)). The current pulse immediately decays and the current intensity returns to its initial value. This transient current is likely photocurrent, and not related to the switching behavior observed with light at 600 and 700 nm. One possible origin of the transient current is the detrapping of charges from the AuNPs adsorbed on the electrode surface far from the junction, since AuNPs have an absorption peak at around 500 nm due to localized surface plasmon resonance^36^.

**Figure 3:**
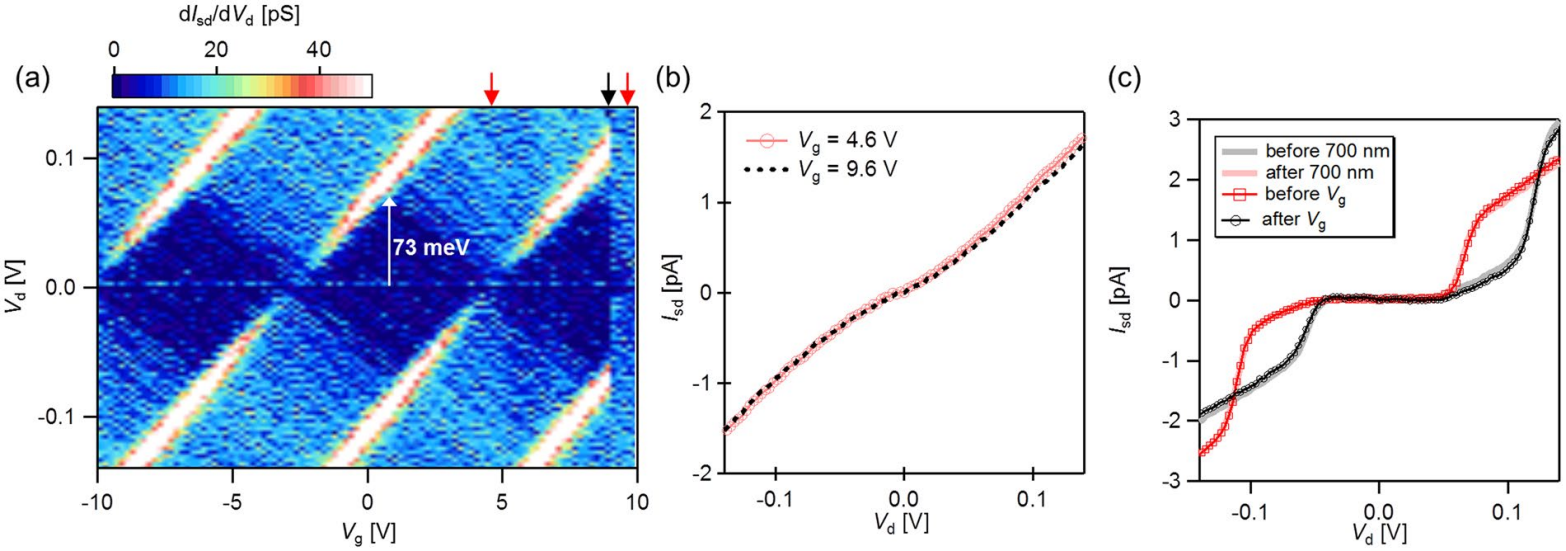
(**a**) Stability diagram of the MFG–SET after light irradiation. (**b**) Comparison of the *I*_sd_ − *V*_d_ characteristics before and after *V*_th_. (**c**) Comparison of the *I*_sd_ − *V*_d_ curves before and after 700 nm light irradiation and applied *V*_g_.

Figure 3(a) shows a stability diagram of the device at state B. A uniform Coulomb diamond structure periodically appears with a charging energy (*E*_c_) of 73 meV, indicating that a single AuNP functions as a Coulomb island. The Coulomb diamond pattern suddenly changes at *V*_g_ = 8.8 V, indicated by the black arrow in Fig. 3(a). This change is not attributed to the breakdown of the device, but to the device state switching from state B to state A. Figure 3(b) shows *I*_sd_ − *V*_d_ curves at *V*_g_ = 4.6 and 9.6 V, giving a comparison of the *I*_sd_ − *V*_d_ curves before and after the discontinuous change in the stability diagram. The *I*_sd_ − *V*_d_ curve at an applied *V*_g_ of 4.6 V corresponds to that at an applied *V*_g_ of 9.6 V, indicating gate offset is shifted by + 5.0 V. The gate-offset shift agrees precisely with the photoinduced gate-offset shift from state A to state B (see Fig. 2(b)). We further confirm this reversible switching behavior by comparing the *I*_sd_ − *V*_d_ curves before and after the switching events. Figure 3(c) shows the *I*_sd_ − *V*_d_ curve measured before and after light-induced switching with and without the application of a high gate voltage. The *I*_sd_ − *V*_d_ curve before applying a high gate voltages corresponds to that after light irradiation, and vice versa. We note that such reversible switching behavior did not appear in the electrical transport characteristics of the device before ttbCuPc deposition. The observation of the reversible change between the two discrete states strongly suggests that this switching behavior originates from the single-electron charging of an individual molecule.

We estimate the efficiency of the floating-gate effect *η* according to the model (Equation (12)), where the period of Coulomb oscillation (Δ*V*_g_) and Δ*V*_mfg_ are obtained from the stability diagram. We determine Δ*V*_g_ = 7.4 V from the Coulomb diamond characteristics (Fig. 3(a)). Conversely, the photoinduced gate offset shift $({\rm{\Delta }}{V}_{{\rm{mfg}}}^{^{\prime} })$ is +5.0 V for the switching from state A to state B. Substituting these values to Eq. (12), we find *η* to be 0.68 in the device leading to a potential shift of over *E*_c_/2 in the Coulomb island.

We discuss here the possible charge states of ttbCuPc corresponding to states A, B, and C. First, the number of molecules contributing to the switching events should be considered. Since there can be several molecules on AuNP, multiple molecules possibly function as a floating gate. If two different ttbCuPc molecules are active during the switching event, two charge states for each molecule are required to exhibit three device states. Conversely, if only one molecule contributes to the switching events, there should be three charge states, such as anion, neutral, and cation, or neutral, cation, and divalent cation. However, contribution of two different molecules is unlikely because the gate offset shift observed for the switching between states A and B is exactly the same as that between states B and C, indicating the same floating gate efficiency (*η*) for the two different ttbCuPc molecules. Since *η* depends on the capacitance between ttbCuPc and AuNP (Equation (4)), atomic scale structural fluctuations are directly reflected in the variation in *η*. In addition, state C is observed only during 700-nm light irradiation as superimposed on state B, while state B is also observed with 600-nm light irradiation, and the lifetime is much shorter than that of state B. If two different molecules with similar gate efficiency contribute to the switching, they would similarly respond to the light irradiation, because their local situation should be similar. Thus, we deduced that single ttbCuPc originates device states B and C.

Copper phthalocyanine is generally considered to be an electron-donating molecule, as used in organic solar cells^37^. It is plausible that CuPc is positively charged due to light absorption, which corresponds to the charge state changing from neutral to cation state. Conversely, Swart *et al*. reported a scanning tunneling microscopy study on the charge state bistability between the anion and neutral states of individual CuPc molecules at 5 K^34^. Borghetti *et al*. reported that a CuPc layer on Ag(111) is charged negatively, while that on Au(111) remains neutral^38^. Although the charge state of the molecules and their stability strongly depend on the energy alignment and atomic scale details at the junction, three charge states of CuPc, *i*.*e*., anion, neutral, and cation, have been reported, which can cause the observed three device states A, B, and C, respectively. Further investigations on the energy-level alignment at ttbCuPc/alkanethiols/AuNP junctions and their photoresponses as well as MFG-SET characteristics with different molecules are required to reveal further details regarding the physics underpinning these devices.

## Conclusion

We demonstrated the photoinduced switching behaviors of ttbCuPc/AuNP-SET, where the individual ttbCuPc molecules function as floating gates and the charge states of ttbCuPc molecules are responsible for the device state. The observed switching between states A and B was completely reversible, and both states were sufficiently stable without external stimuli. Based on the observation of clear Coulomb diamonds and photoinduced gate offset shifts, we derived the MFG efficiency *η* of the device to be 0.68. This value indicates that the amount of potential shift induced by single-electron charging of ttbCuPc exceeds *E*_c_/2, which suggests that the effect is sufficiently large for logic and memory operation using the SETs. Unfortunately, at this stage, it is still difficult to obtain enough number of devices for statistical analysis and control the floating-gate efficiency. However, it is worth mentioning the importance of such high efficiency in term of the use of single molecule as a functional material in nanoscale devices, though the estimated floating-gate efficiency is specific to the present device. Remarkably, an additional device state (state C) was observed during 700-nm light irradiation. This state agreed well with that expected for doubly charged ttbCuPc. These results clearly demonstrate a unique function of an SET that originates from the charge state of an individual molecule, which means that MFG-SETs can be candidates for alternative molecular electronics devices without the typical molecular junctions and used as tools for investigating molecular charging phenomena.

## Methods

### Fabrication process

Nanogap electrodes were fabricated using an electroless gold-plating method^39, 40^. The initial Au (10 nm)/Ti (2 nm) electrode patterns were fabricated on a SiO_2_/Si substrate by using electron beam lithography and a lift-off process. The substrate was immersed in an Au iodine solution, and the gap width was reduced via a self-termination reaction.

In order to define the contact between the AuNP and the electrodes, the electrode surface was modified with a mixed self-assembled monolayer (SAM)^41^. Nanaogap electrodes were successively immersed into a hexanethiol solution (1 mM, ethanol) and octanedithiol solution (1 mM, ethanol) for 12 h each. The nanogap electrodes were finally immersed in a AuNP solution for 12 h. AuNPs were then absorbed on the nanogap electrodes through the octanedithiols. The substrate was then transferred to the evaporation chamber and ttbCuPc molecules were deposited with a density of ~0.9 molecules/nm^2^. This density corresponds to about 7 molecules per one AuNP.

### Transport measurements

The electrical measurements^23–26^, current–time (*I*_sd_ − *t*) and *I*_sd_ − *V*_d_ − *V*_g_ measurements, were performed before and after molecular depositon using a low temperature vacuum probe station at a temperature of 9 K. The incident light source was a xenon lamp where the wavelength was selected via band pass filter. The device was illuminated through a view port. The wavelength and intensity of the incident lights were 700 nm (280 *μ*W/mm^2^), 600 nm (52 *μ*W/mm^2^), 520 nm (128 *μ*W/mm^2^), and 500 nm (79 *μ*W/mm^2^), respectively.
